# Developing emotional intelligence and counseling self-efficacy in clinical pastoral education in healthcare settings: a multicenter pre-post study

**DOI:** 10.3389/fmed.2025.1578653

**Published:** 2025-10-10

**Authors:** Csaba Szilagyi, Patricia K. Palmer, Paul Galchutt, Kristin Langstraat, George Fitchett

**Affiliations:** ^1^College of Health Sciences, Rush University, Chicago, IL, United States; ^2^Woodruff Health Sciences Center, Emory University, Atlanta, GA, United States; ^3^TriHealth, Cincinnati, OH, United States

**Keywords:** emotional intelligence, counseling self-efficacy, chaplaincy education, clinical pastoral Education, health professions education, spiritual care, chaplaincy

## Abstract

**Introduction:**

Emotional intelligence (EI) and counseling self-efficacy (CSE) are essential qualities for healthcare chaplains who support individuals experiencing emotional and spiritual distress. Clinical Pastoral Education (CPE) serves as the primary clinical learning environment for developing EI and counseling skills within spiritual care practice. While limited research suggests CPE’s positive effects on personal and professional development, existing studies have methodological limitations. This study addressed these gaps by using validated instruments within a multicenter, pretest-posttest design. We investigated changes in EI and CSE during CPE, explored predictive factors, and examined the relationship between changes in EI and CSE among CPE students participating in their initial CPE unit (i.e., interns) and those in CPE residency programs (i.e., residents).

**Methods:**

We used the Schutte Self-Report Emotional Intelligence Test and the Counselor Activity Self-Efficacy Scales to assess changes in EI and CSE. Students at ACPE-accredited CPE centers across the United States completed pre- and post-training surveys, along with mid-residency surveys for residents. Statistical analyses evaluated changes over time, compared interns and residents, and explored relationships between EI and CSE. Linear regression models identified significant predictors of pre-to-post changes.

**Results:**

Our sample included 196 participants (mean age = 45.08 years; 47.4% female) from 29 accredited CPE centers, representing 9.6% of all centers across the United States. Most participants (95.9%) completed their training in healthcare settings. Significant increases in EI and CSE were observed among both CPE interns and residents (*p* < 0.001), with moderate to large effect sizes for EI and CSE. EI gains significantly predicted CSE improvements for both groups, with moderate correlations between EI and CSE changes. More CPE (residents vs. interns) facilitated greater increases in outcome variables, and longer program duration predicted greater EI gains for interns. The mode of delivery—in-person, hybrid, or online—did not significantly impact EI and CSE gains.

**Conclusion:**

This study demonstrates that participants’ EI and CSE significantly increased during their clinical education. The results show a strong relationship between EI and CSE development, suggesting that EI growth contributes to increased confidence in counseling abilities. The findings support CPE’s effectiveness in fostering essential skills for spiritual care providers, regardless of delivery modality or participant demographics.

## 1 Introduction

Healthcare chaplains, as spiritual care specialists, provide assessment, counseling, and advocacy to support individuals in coping with their illness and healthcare experiences (1–4). Counseling in spiritual care entails helping people of all backgrounds use their spiritual, religious, value-system, meaning-making, emotional, social, cultural, and personal resources in difficult situations in light of their lived experiences and health (distinct from providing psychotherapy or mental health treatment) (1, 2, 4). Chaplains care for patients and their families when they face existential, spiritual, or emotional distress, crisis, trauma, ethical dilemmas, medical decision-making, end-of-life, or loss (1, 3, 4). To function effectively in these situations, chaplains need to recognize, understand, and manage emotional dynamics in self, others, and relationships, while applying effective helping and counseling skills (3).

Growing evidence underscores the need for and the impact of integrating spiritual care into healthcare. A recent systematic review in *JAMA* recommended integrating routine spiritual care and spiritual care specialist chaplains in the healthcare of individuals with serious illnesses (5). Synthesis of the evidence showed that religion and/or spirituality and spiritual care are essential for coping with illness and health outcomes. Religion and/or spirituality are important to most patients with serious illness, who commonly report spiritual needs. Patients often desire spiritual care that improves their quality of life. Chaplaincy services and interventions were associated with a range of positive quality of life, spiritual and psychological wellbeing, and patient satisfaction outcomes in inpatient and outpatient settings (6, 7).

Clinical Pastoral Education (CPE) is the primary method of clinical learning for chaplains-in-training to develop their emotional intelligence (EI) and counseling skills for spiritual care practice. CPE is the standard clinical training in spiritual care and chaplaincy that is required for becoming a board-certified healthcare chaplain by major certifying organizations in the United States (8–12). CPE uses experiential and action-reflection learning methods. CPE students learn from clinical experiences with the diverse individuals they serve, integrated with clinical supervision, critical reflection, verbatim case discussions and feedback, and didactic instruction in the contexts of the peer group and the supervisory alliance with their educator (12–14). In the United States, the ACPE: The Standard for Spiritual Care and Education (ACPE) (formerly the Association for Clinical Pastoral Education) is the premier accreditor ensuring adherence to established standards and learning objectives for accredited CPE programs (12, 15). ACPE certifies ACPE educators as qualified to provide CPE training and lead CPE groups (16). Generally, CPE objectives aim to foster personal and professional integration and growth and train students to be competent in providing spiritual care for individuals and groups.

The limited research on the impact of CPE generally shows personal, professional, and clinical skills development in areas relevant to our study. An early pretest-posttest study showed that CPE improved students’ abilities in the areas of counseling resources, facilitative relations, problem resolution, and non-judgmental acceptance (17). In a post-training survey, CPE students reported that they learned about their personal functioning, the group process helped them articulate their feelings, they became more aware of their abilities as chaplains, and the training helped them understand relationship dynamics (18). A review of quantitative studies of CPE summarized the evidence for positive changes in actualizing students’ “human potential,” beliefs in succeeding in ministry, and future performance in ministry (19).

More recently, Jankowski et al. (20) found increases in EI, self-reflection, and pastoral skills over a single CPE unit in one CPE center. In a recent qualitative study, graduates of CPE reported lasting effects of CPE in areas including self-awareness, emotional intelligence, personal growth, and other awareness, as well as in communication, listening, interventions, and assessment skills (21). A pre-post mixed methods study found that CPE students developed their EI, counseling self-efficacy (CSE), and listening and attending skills during a CPE unit (14). Overall, the weaknesses in existing CPE research include small sample sizes drawn from one or only a limited number of CPE programs, predominantly cross-sectional designs, and participants recruited from the researchers’ own students and programs, increasing the risk of bias (19). More pretest-posttest multicenter studies with larger samples that investigate measurable outcomes with standardized instruments are needed.

Developing and using EI and counseling skills are critical for chaplains to help people deal with spiritual and emotional distress. This involves assisting individuals in coping, improving their wellbeing, finding meaning, making decisions, reflecting on emotions, relationships, and the transcendent, and actively listening to them. Our study adds to the literature by using a multicenter repeated-measures design and validated instruments to measure changes in two key outcome variables: emotional intelligence and counseling self-efficacy.

Emotional intelligence is a vital construct to investigate in chaplaincy education and practice because EI involves the critical capacity to understand and manage one’s emotional world and use emotions as resources for making sense of and navigating one’s social interactions (22). Our work primarily relied on the Salovey and Mayer (23) EI model and its refined four-branch version (24). It posits that EI includes four increasingly complex areas with corresponding abilities to (a) accurately perceive and express emotion, (b) use emotion to facilitate thinking (by generating emotions to facilitate thought and tailoring thinking to emotion), (c) understand emotions and their meanings, and (d) manage emotions in self and others (24, 25).

Few prior studies have examined EI in chaplaincy education and practice despite the potential benefits that improving EI would have on chaplain clinical practice. Dissertation research by Makau-Olwendo (26) showed that chaplains readily employed EI in their clinical interventions in CPE and their ongoing practice. Szilagyi et al. (14) found that increasing EI during CPE enhanced chaplains’ ability to provide care in emotionally laden clinical encounters, paralleling other studies showing that CPE fostered EI (20, 21). In other health professions, practical and EI training have been shown to improve a range of EI domains and their correlates (27–29).

Regarding EI-related factors among healthcare chaplains, dissertation research by Nomsule (30) found positive relationships between EI and job satisfaction, which is congruent with EI’s positive impact on work-related psychological health by reducing emotional exhaustion and increasing job satisfaction found among clergy (31). Furthermore, among a range of health professionals, the evidence shows that EI has been positively associated with caring behavior (32, 33), compassion satisfaction (34), clinical decision-making (35), and leadership qualities (27) and negatively associated with compassion fatigue (34) and emotional exhaustion and burnout (33, 36).

Overall, the evidence highlights the benefits of developing EI for direct patient care and professional wellbeing in healthcare. However, the construct of EI has not been widely studied in the field of chaplaincy practice and education. Our study sought to examine whether EI improves with CPE participation and what demographic and educational factors predict such improvement.

Counseling self-efficacy refers to the belief in one’s own abilities to effectively counsel clients in the context of their practice (37). This well-established construct encompasses individuals’ confidence in performing helping skills and counseling-related behaviors and managing client relationships and issues successfully. Counseling self-efficacy impacts counselors’ responses, emotions, cognition, and performance. Research has identified positive correlations between CSE and various factors, including counselor preparation and career development (38, 39), helping and counseling skills training (40, 41), practicum experiences (involving direct services and supervision) (38, 42, 43), and counselors’ cognitive and affective experiences (38, 40, 44). Regarding client outcomes, studies have found an inverse session-to-session relationship between CSE and client distress (with CSE mediating working alliances between counselors and clients) (45) and an association between CSE gains and client symptom improvement (43).

Counseling self-efficacy is a crucial factor in training because it may foster and reflect individuals’ motivation and abilities to use counseling skills and manage counseling processes and relationships effectively. Self-efficacy functions as a bridge between knowledge and action (and between training and practice) as CSE is “a primary mechanism between simply knowing how to help in a counseling situation and actually executing effective counseling actions” (44). Thus, self-efficacy helps counselors integrate their acquired cognitive, social, and behavioral skills to persistently carry out complex courses of action in dynamic environments (37, 46). Counseling self-efficacy and changes in CSE have been investigated among trainees and practitioners of various helping professions, such as counseling (38, 39, 42), social work (40), psychology (47), and speech-language pathology (48), and among undergraduates receiving helping skills training (41). These studies demonstrate positive relationships between CSE and counseling courses, practical/clinical training, and clinical experience. However, to date, CSE and its development have not been investigated in CPE or chaplaincy, except for a small-scale evaluation study by Szilagyi et al. (14). Overall, evidence indicates that CSE is essential when developing helping skills and counseling competencies and offers a valid self-reported estimation for measuring baseline and gains in skills, particularly in translating knowledge to effective action in practice.

We posit that EI and CSE are fundamental and expected in the role of the chaplain. Furthermore, based on the research showing their positive association (49), we theorize that EI and CSE are interconnected, EI influences CSE, and they are essential for chaplaincy practice. This is particularly prominent in seeing that basic EI capabilities of perceiving one’s own and others’ emotions and utilizing emotion to think and understand others (25) influence social interactions and aid one’s use of counseling skills in helping others. This is supported by research demonstrating significant positive correlations between CSE and identifying emotions in self and others, managing others’ emotions, and using emotions to problem-solve (49). Mand et al. (48) found that EI, along with training and years of experience, promoted CSE among speech-language pathologists. These interlinked constructs have vital implications for chaplaincy practice. When chaplains are exposed to a wide range of intense emotions and human suffering in complex health-related situations, they need to function as emotionally intelligent, caring professionals, understand and manage emotions, and perform helping and counseling behaviors effectively. Although learning and using EI and helping skills are considered hallmarks of CPE and spiritual care practice, their development and apparent links warrant a more thorough examination.

Our study’s main objective was to investigate the changes in EI and CSE among CPE students participating (a) in their first stand-alone unit of CPE (i.e., interns) and (b) in CPE residency programs (i.e., residents). We hypothesized that EI and CSE increase over the course of CPE in both subgroups. In addition, we aimed to identify potential demographic and educational factors that may predict changes in EI and CSE among both types of CPE students. We also sought to examine the relationships between changes in EI and CSE. In the exploration of predictors, we anticipated that improvement in EI would predict CSE gains.

## 2 Materials and methods

We used a quasi-experimental research design with repeated-measures surveys. This report specifically focuses on the survey phase of a larger, comprehensive research project utilizing mixed methods. The protocol for this research was deemed exempt by the Johns Hopkins Medicine Institutional Review Board (IRB00252823). We obtained informed consent from all participants.

### 2.1 Participants

We recruited Clinical Pastoral Education students enrolled at ACPE-accredited CPE centers nationwide in the United States. CPE students who were enrolled (a) in their first stand-alone unit of CPE (also known as “internship”) or (b) in a CPE residency program (typically including three or four consecutive CPE units) were eligible. For this report, we refer to the first subgroup, the first-time CPE students, as “interns.” We use the term “resident” for the second category of CPE students in our sample. Students enrolled at a center not accredited by ACPE and interns completing their second or subsequent CPE units were ineligible. We partnered with ACPE Certified Educators, who responded to email announcements about the study and allowed us to recruit students at their CPE centers. These educators reported the number of eligible students enrolled and the start and end dates of their CPE programs. We emailed invitations—including study information and a link to consent and survey—to CPE educators who distributed them to their students during the respective survey windows. CPE students choose to participate voluntarily based on the study information, without incentives given. Educators had no access to survey responses. An *a priori* power analysis for sample size was not conducted because it is unnecessary in cohort studies where the entire sampling frame is invited to participate.

### 2.2 Procedures

All participants undertook CPE at an ACPE-accredited CPE center. All CPE units were led according to ACPE Standards and Outcomes (12) by educators certified by ACPE or certified educator candidates under the supervision of an ACPE Certified Educator. We did not require experimental interventions nor departures from the CPE programs’ already established educational practices or curricula. Overall, our procedures supported the study’s objective to examine changes associated with CPE as typically delivered.

According to ACPE Standards, one CPE unit comprises 400 hours of training, which combines a minimum of 100 hours of structured group and individual instruction and a minimum of 250 hours of supervised clinical practice in spiritual care (50, 51). Interns in our study completed one CPE unit without any prior CPE training. Residents typically completed 3 or 4 consecutive CPE units over a period of 8 to 12 months, with a general prerequisite of at least one initial CPE unit before entry.

### 2.3 Data collection

All participants completed pre- and post-training surveys at the beginning and end of their CPE program. In addition, residents completed a mid-residency survey halfway through their program. Since all CPE student groups had different start and end dates at their institutions during the study, we aligned data collection points with each group’s dates. Pre-training surveys occurred within the first 2 weeks, and post-training surveys occurred within the last 2 weeks of CPE training. The 2-week windows of the mid-residency surveys were centered at the calendar-day middle point of each residency program. Table 1 shows the survey measurements used at each data collection point. For anonymous data collection and matching longitudinal data, we had participants use self-generated study identification codes. Self-generated identification codes are commonly and effectively used to link pre-post data, assure participants of their anonymity, and protect personally identifiable information with low incorrect match rates (52, 53). Survey data were collected online using Research Electronic Data Capture (REDCap), a secure, web-based software platform hosted at Johns Hopkins University (52, 53). We gathered data between July 2020 and January 2022 (54).

**Table 1:** Data collection points and survey measurements.

Data collection points	Subgroups	Measurements
Pre-training survey at the start of the CPE program	Interns Residents	Demographic Questionnaire Emotional Intelligence (SSEIT) Counseling Self-Efficacy (CASES)
Mid-residency survey midway through the CPE residency program	Residents	Emotional Intelligence (SSEIT) Counseling Self-Efficacy (CASES)
Post-training survey at the end of the CPE program	Interns Residents	Emotional Intelligence (SSEIT) Counseling Self-Efficacy (CASES) Educational Factors Questionnaire

CPE, Clinical Pastoral Education; SSEIT, Schutte Self-Report Emotional Intelligence Test (55, 56); CASES, Counselor Activity Self-Efficacy Scales (38).

### 2.4 Emotional intelligence

The Schutte Self-Report Emotional Intelligence Test (SSEIT) (also called the Assessing Emotions Scale) assessed respondents’ characteristic adaptive emotional functioning (55, 56). Developed by Schutte et al. (55) based on the Salovey and Mayer EI model (24), the 33-item instrument was designed to measure how well individuals typically appraise emotions in self and others, express emotions, regulate emotions in self and others, and utilize emotions. Participants rated the extent to which each statement accurately described them on a 1–5 scale, where 1 meant Strongly disagree, and 5 meant Strongly agree. After reverse coding three items, the total score resulted from the sum of all items (range 33–165), with higher scores representing higher EI. Similar to its validation study (55), the SSEIT had strong internal consistency in the current study’s sample, with a baseline Cronbach’s alpha (CA) = 0.87, mid-year CA = 0.86, and post-CA = 0.89.

Schutte et al. (56) recommended the following subscales, based on factor analysis by Ciarrochi et al. (57): Perception of Emotion (10 items), Managing Own Emotions (9 items), Managing Others’ Emotions (8 items), and Utilization of Emotion (6 items). Although others identified variation in factor loadings, which suggests subscales should be used with some caution (58, 59), we examined our data using these recommended subscales. Subscales ranged from very good to adequate internal consistency in our sample at baseline (CA = 0.81, 0.77, 0.57, and 0.66, respectively), mid-year (CA = 0.78, 0.71, 0.54, and 0.64, respectively), and post-training (CA = 0.83, 0.78, 0.62, and 0.77, respectively). We reported subscale results as the average item rating to allow for comparing subscales with differing numbers of items. Using the SSEIT instrument also enabled direct comparisons with other studies measuring EI changes in CPE (14, 20).

### 2.5 Counseling self-efficacy

The Counselor Activity Self-Efficacy Scales (CASES) Part 1 and Part 2, developed by Lent et al. (38), measured CSE. The 15-item Part 1: Helping Skills Self-Efficacy scale (our sample’s baseline CA = 0.91, mid-year CA = 0.93 and post-training CA = 0.92) asked participants to rate their confidence in their ability to perform helping and counseling skills effectively in three subscales: (a) Insight Skills to help clients better understand their thoughts and feelings (6 items; baseline CA = 0.83, mid-year CA = 0.89, and post-training CA = 0.86), (b) Exploration Skills to ask open questions, listen, reflect feelings, restate, and attend (5 items; baseline CA = 0.87, mid-year CA = 0.89, and post-training CA = 0.87), and (c) Action Skills to employ more structured counseling interventions (4 items; baseline CA = 0.85, mid-year CA = 0.84, and post-training CA = 0.88). The 10-item Part 2: Session Management Self-Efficacy scale assessed perceived capabilities to keep sessions focused and help clients understand and talk about their concerns at a deeper level (10 items; CA = 0.95, mid-year CA = 0.95, and post-training CA = 0.93). The internal consistency of CASES in our sample was consistent with the instrument’s validation study (38). We did not use CASES Part 3: Counseling Challenges because it assesses skills specific to challenges in mental health counseling distinct from chaplains’ scope of practice. Respondents rated their self-efficacy on a 0–9 scale, where 0 meant “No Confidence,” and 9 meant “Complete Confidence.” The average item score was calculated for scales and subscales. Higher scores reflected greater confidence in one’s counseling capabilities.

### 2.6 Demographic and educational factors

The baseline surveys gathered information about participants’ demographics, such as age, gender, ethnicity, religious or spiritual background, and level of education, in addition to their prior work experience relating to spiritual care or counseling and general professional backgrounds. Residents were also asked about the number of prior CPE units they had completed, if any. The post-training surveys included a questionnaire about CPE educational factors, such as delivery modality and clinical context. Interns were also queried on the length of their CPE unit.

### 2.7 Statistical analysis

Descriptive statistics (mean and standard deviation for numerical variables, frequency and percentage for categorical variables) were used to characterize baseline demographics and CPE setting characteristics. Where participants entered a description in an available “other” category that was identical or equivalent to a named category, they were counted in the named category; for example, an entry of “Baptist” in the other religion category was counted as Protestant. Expectation maximization was used to generate missing values using the other items in the scale (SSEIT) or subscale (CASES) as predictors. Little’s Missing Completely at Random test was used to confirm that values were missing at random. Missing values were less than 5% of the total data in all cases. Negatively phrased EI items were reverse-scored prior to the calculation of missing values or total scale and subscale scores. Average item rating scores were calculated to allow comparison of SSEIT and CASES subscales having different numbers of items. The normality of outcome variables was evaluated using the Shapiro–Wilk test.

We described SSEIT and CASES scores across time using mean and standard deviation and used paired samples *t*-tests (when differences were normally distributed) or Wilcoxon signed-rank test (where differences were non-normal) to evaluate changes in outcome measures across the course of the CPE internship or residency (pre-training to post-training). Effect sizes are reported as Cohen’s d for the *t*-test, and as rank serial correlation r for the Wilcoxon signed-rank test. Similarly, we used an independent *t*-test (normal data) or Mann–Whitney U test (non-normal data) to compare pre-to-post differences between interns and residents. Spearman’s correlation test was used to explore correlations between the SSEIT and CASES scales.

Linear regression was used to identify significant predictors of pre-to-post change over the course of a CPE internship or residency program for three outcome measures: SSEIT, CASES Part 1: Helping Skills Self-Efficacy, and CASES Part 2: Session Management Self-Efficacy scale. Covariates in the regression included available demographics (age and gender) as well as theory-based variables, including years of relevant spiritual care or counseling experience prior to CPE; length of the program (for interns only, 20 weeks or more for a part-time unit compared to a shorter-duration intensive or extended unit); and method of program delivery (in-person, on-line, or hybrid). Because EI theoretically precedes CSE, the pre-to-post CPE change in SSEIT was added as a covariate to the CASES Part 1 and Part 2 regressions. Ethnicity, religion, and education were not included due to insufficient participants in subcategories and to keep the regression model parsimonious. To confirm the appropriateness of linear regression, the normality of the dependent variables was evaluated using the Shapiro–Wilk test. Pre-to-post CASES Part 2 gain among residents was the only non-normally distributed outcome; therefore, the outcome was Log_10_ transformed before performing the regression. After transformation, regression residuals were confirmed to be normally distributed, confirming the applicability of linear regression. *Post-hoc* comparisons of baseline levels were conducted for predictors and outcomes of interest identified in the regressions.

## 3 Results

### 3.1 Participant characteristics

**Figure 1:**
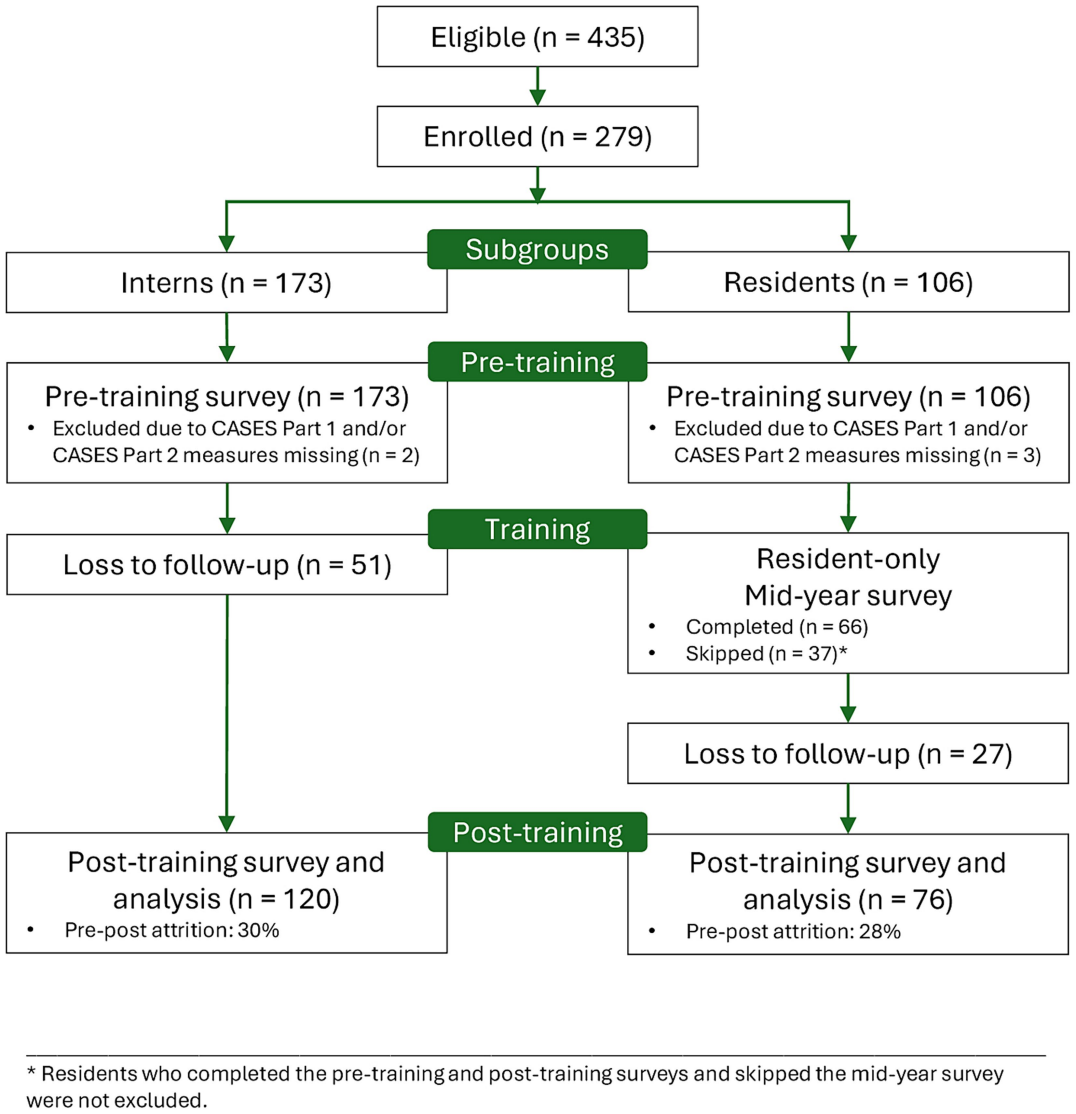
Diagram showing the flow of participants through the pre-post study.

A total of 279 individuals submitted the baseline survey, consisting of 173 (62%) first-unit CPE interns and 106 (38%) CPE residents. Participating CPE programs reported 435 eligible CPE students in their respective CPE programs, which yielded a 64% baseline response rate. The post-training surveys were completed by 196 participants, 72% of those at baseline. The attrition consisted of students who did not complete the post-training survey or completed it only partially and students who withdrew from their respective CPE training and thus became ineligible. Figure 1 presents the flow of participants and attrition throughout the study. Our analysis focused on 196 respondents with matched pre-post data, including 120 (61%) first-unit CPE interns and 76 (39%) CPE residents. These participants were enrolled in CPE at 29 ACPE-accredited centers in 15 states of the United States, with all four U. S. census regions represented (i.e., Northeast, Midwest, South, and West). The 29 centers represented 9.6% of the 303 ACPE-accredited centers in 2020 in the United States (60).

Table 2 presents the sample’s demographic data. Participants’ mean age was 45.08 (SD = 12.96) years. Half the sample (50%) was male. Sixty-three percent identified as white, 18.9% as Black, and 6.6% as Asian. Most participants described their religious background as Protestant (47.4%), Other Christian (25.0%), and Catholic (14.3%). Half the sample (50.0%) reported more than 3 years of prior work experience related to spiritual care, counseling, or other caregiving/helping roles.

**Table 2:** Participant characteristics.

Characteristic	Total	CPE interns	CPE residents
	*n* (%)	*n* (%)	*n* (%)
Age (mean, SD)	45.08 (12.96)	45.47 (13.27)	44.49 (12.53)
Gender			
Men	98 (50.0)	63 (52.5)	35 (46.1)
Women	93 (47.4)	54 (45.0)	39 (51.3)
Non-binary/third gender	2 (1.0)	1 (0.8)	1 (1.3)
Prefer not to say	2 (1.0)	1 (0.8)	1 (1.3)
Missing	1 (0.5)	1 (0.8)	
Ethnicity			
Not Hispanic/Latino	186 (94.9)	113 (94.2)	73 (96.1)
Hispanic/Latino	10 (5.1)	7 (5.8)	3 (3.9)
Race			
White	124 (63.3)	73 (60.8)	51 (67.1)
Black	37 (18.9)	24 (20.0)	13 (17.1)
Asian	13 (6.6)	9 (7.5)	4 (5.3)
Mixed ethnicity	9 (4.6)	7 (5.8)	2 (2.6)
Other/Prefer not to say	11 (5.6)	6 (5.0)	5 (6.6)
Missing	2 (1.0)	1 (0.8)	1 (1.3)
Religious or spiritual background			
Protestant	93 (47.4)	61 (50.8)	32 (42.1)
Other Christian	49 (25.0)	29 (24.2)	20 (26.3)
Catholic	28 (14.3)	18 (15.0)	10 (13.2)
Jewish	5 (2.6)	1 (0.8)	4 (5.3)
Buddhist	3 (1.5)	0 (0.0)	3 (3.9)
Spiritual, not religious	3 (1.5)	2 (1.7)	1 (1.3)
Orthodox Christian	3 (1.5)	2 (1.7)	1 (1.3)
Muslim	1 (0.5)	0 (0.0)	1 (1.3)
Other Eastern religion/spirituality	1 (0.5)	1 (0.8)	0 (0.0)
Neither religious nor spiritual	1 (0.5)	0 (0.0)	1 (1.3)
Other, prefer to self-describe^a^	8 (4.1)	5 (4.2)	3 (3.9)
Missing	1 (0.5)	1 (0.8)	
Related prior work experience (years)			
None	29 (14.8)	27 (22.5)	2 (2.6)
Less than 1 year	18 (9.2)	9 (7.5)	9 (11.8)
1 to 3 years	51 (26.0)	28 (23.3)	23 (30.3)
More than 3 years	98 (50.0)	56 (46.7)	42 (55.3)
Type of related prior work experience			
Faith leader/clergy	119 (60.7)	70 (58.3)	49 (64.5)
Spiritual care/chaplaincy	53 (27.0)	20 (16.7)	33 (43.4)
Community/social services organization	28 (14.3)	16 (13.3)	12 (15.8)
Healthcare	22 (11.2)	13 (10.8)	9 (11.8)
Counseling	16 (8.2)	9 (7.5)	7 (9.2)
Other^b^	26 (13.3)	19 (15.8)	7 (9.2)
General professional background			
Graduate student or seminary student	73 (37.2)	46 (38.3)	27 (35.5)
Faith leader/clergy	57 (29.1)	32 (26.7)	25 (32.9)
Chaplain	16 (8.2)	8 (6.7)	8 (10.5)
Other	50 (25.5)	34 (28.3)	16 (21.1)
Delivery of CPE			
In-person	82 (41.8)	53 (44.2)	29 (38.2)
Hybrid	64 (32.7)	27 (22.5)	37 (48.7)
Online	45 (23.0)	35 (29.2)	10 (13.2)
Missing	5 (2.6)	5 (2.6)	0 (0.0)
Length of CPE unit (Interns only)			
10–12 weeks (intensive)		42 (35.0)	n/a
12–19 weeks (extended)		45 (37.5)	n/a
20 or more weeks (part time)		29 (24.2)	n/a
Missing		7 (3.6)	n/a
Number prior CPE units (Residents only)			
0 units		n/a	9 (11.8)
1 unit		n/a	57 (75.0)
2 units		n/a	5 (6.6)
3 units		n/a	2 (2.6)
4 or more units		n/a	3 (3.9)
Setting of CPE training			
Hospital	170 (86.7)	96 (80.0)	74 (97.4)
Hospice	17 (8.7)	16 (13.3)	1 (1.3)
Parish/faith-based communities	5 (2.6)	5 (4.2)	0 (0.0)
Long-term care facility	1 (0.5)	0 (0.0)	1 (1.3)
Missing	3 (1.5)	3 (1.5)	0 (0.0)

CPE, Clinical Pastoral Education; SD, standard deviation.

a Other religion/spirituality included Unitarian Universalist (*n* = 5); Buddhist-Christian (*n* = 1); Interfaith (*n* = 1); and Humanist Confucian (*n* = 1).

b Other category-related work included education, work in non-profit organizations, yoga, and coaching. Four participants also named non-related work experiences, including roles as a student, truck driver, plumbing, and paralegal.

### 3.2 Changes in emotional intelligence

There were statistically significant increases in EI across their respective CPE programs among interns and residents, with moderate effect sizes for SSEIT and its subscales (Table 3). At baseline, interns reported a mean EI score of 126.44 (SD = 12.22) on SSEIT. At the end of their CPE, interns’ EI score significantly improved to 130.99 (*p* < 0.001), a 3.6% improvement. As detailed in Table 3, there was statistically significant growth in all EI subscales. Increases were similar in magnitude, with the highest pre-post rate of increase on the Utilization of Emotion (4.0%) subscale and the lowest growth on the Perception of Emotion (2.9%).

**Table 3:** Changes in emotional intelligence and counseling self-efficacy in Clinical Pastoral Education (CPE).

Measure	Interns (mean, SD) *n* = 120					Residents (mean, SD) *n* = 76						Interns vs. residents gain
	Pre	Post	% Δ	Effect size	*p* ^a^	Pre	Mid (*n* = 66)	Post	% Δ	Effect size	*p* ^a^	*p* ^b^
Emotional intelligence (EI)												
Overall EI (SSEIT; range: 33–165)	126.44 (12.22)	130.99 (13.06)	3.6	−0.44	<0.001	127.22 (12.56)	130.02 (10.72)	135.05 (11.08)	6.2	−0.59	<0.001	0.057
Overall EI (average SSEIT 1–5 item rating)^c^	3.83 (0.37)	3.97 (0.40)	3.6	−0.44	<0.001	3.86 (0.38)	3.94 (0.32)	4.09 (0.34)	6.2	−0.59	<0.001	0.057
Perception of Emotion^c^	3.79 (0.50)	3.90 (0.53)	2.9	−0.27	0.003^d^	3.82 (0.52)	3.88 (0.44)	4.07 (0.44)	6.5	−0.47	<.001^d^	0.084^e^
Managing Own Emotions^c^	3.84 (0.54)	3.99 (0.50)	3.9	−0.34	<0.001	3.86 (0.42)	3.96 (0.42)	4.11 (0.43)	6.5	−0.64	<0.001	0.091
Managing Others’ Emotions^c^	3.91 (0.41)	4.06 (043)	3.8	−0.37	<.001^d^	3.85 (0.45)	3.89 (0.39)	4.14 (0.39)	7.5	−0.60	<0.001	0.037
Utilization of Emotion^c^	3.79 (0.51)	3.94 (0.53)	4.0	−0.34	<.001^d^	3.91 (0.46)	4.07 (0.46)	4.04 (0.57)	3.3	−0.28	0.016^d^	0.884^e^
Counseling self-efficacy												
CASES Part 1: Helping Skills Self-Efficacy (range: 0–9)	5.75 (1.31)	6.68 (1.15)	16.2	−0.72	<0.001	6.03 (1.10)	6.69 (1.21)	7.23 (1.04)	19.9	−0.74	<.001^d^	0.090
Insight Skills^c^	5.68 (1.52)	6.57 (1.31)	15.7	−0.52	<.001^d^	5.84 (1.24)	6.55 (1.41)	7.11 (1.12)	21.7	−0.68	<.001^d^	0.088
Exploration Skills^c^	6.67 (1.24)	7.59 (0.93)	13.8	−0.61	<.001^d^	7.15 (1.00)	7.60 (1.01)	8.13 (0.79)	13.7	−0.75	<.001^d^	0.482^e^
Action Skills^c^	4.73 (1.94)	5.71 (1.84)	20.7	−0.48	<.001^d^	4.93 (1.71)	5.77 (1.78)	6.28 (1.91)	27.4	−0.58	<.001^d^	0.191
CASES Part 2: Session Management Self-Efficacy (range: 0–9)	5.74 (1.52)	6.80 (1.20)	18.5	−0.66	<.001^d^	6.08 (1.42)	6.87 (1.18)	7.41 (1.04)	21.9	−0.78	<.001^d^	0.204

a Paired *t*-test was used to compare pre-to-post values except where otherwise indicated.

b Independent *t*-test used unless otherwise indicated.

c Scale and subscales reported as the average item rating (1–5 range for SSEIT; 0–9 range for CASES) to allow for comparison between subscales that have differing numbers of items.

d Wilcoxon signed-rank test was used to compare pre-to-post values due to non-normal distribution.

e Mann–Whitney U test was used to compare interns’ vs. residents’ pre-to-post gain due to non-normal distribution.

Δ = Change; SSEIT, Schutte Self-Report Emotional Intelligence Test (55, 56); CASES, Counselor Activity Self-Efficacy Scales (38).

Effect size presented as Cohen’s d for *t*-test, characterized as small (0.2), moderate (0.5), or large (0.8) and as *r* (rank-serial correlation) = Z/√(N) for non-parametric tests characterized as small (< 0.3), moderate (0.3–0.5), and large (> 0.5); the negative effect sizes indicate that post-training scores are higher than pre-training scores.

Residents started at a baseline EI of 127.22 (SD = 12.56) on SSEIT, which increased to 130.02 by the midpoint of residency and reached 135.05 at post-residency (pre-post change 6.2%, *p* < 0.001). It is worth noting that residents’ EI growth did not plateau or slow at the midpoint but rather slightly accelerated as the EI change from mid- to post-residency was greater than from pre- to mid-residency. Residents had the greatest pre-post gains on the Managing Others’ Emotions subscale (7.5%) and the least increase on the Utilization of Emotions subscale (3.3%).

### 3.3 Changes in counseling self-efficacy

There were statistically significant gains in counseling self-efficacy during CPE among both interns and residents, with effect sizes for the CASES scales and subscales ranging from moderate to large (Table 3). The baseline for interns on the CASES Part 1: Helping Skills Self-Efficacy scale was 5.75 (SD = 1.31) and on the CASES Part 2: Session Management Self-Efficacy scale was 5.74 (SD = 1.52). Interns’ CSE significantly increased on all CASES scales and subscales (*p*-values <0.001). Interns had the largest CSE gains on the Action Skills subscale (20.7%), which had the lowest baseline relative to other CASES subscales. Conversely, interns reported the lowest rate of growth on the Exploration Skills subscale (13.8%), which had the highest baseline and post-training levels compared to other subscales.

Among residents, the baseline levels of CSE were 6.03 (SD = 1.10) on CASES Part 1 and 6.08 (SD = 1.42) on Part 2. Scores rose statistically significantly on CASES Part 1 and CASES Part 2 (*p*-values <0.001). Similar to interns, residents gained the most on the Action Skills subscale (27.4%) and the least on the Exploration Skills subscale (13.7%). Again, the subscale with the greatest gain showed the lowest reported baseline level and vice versa. A trajectory similar to their EI changes can be seen over time as residents’ CSE growth did not halt at the midpoint of the residency but continued throughout the duration of their training programs.

### 3.4 Predictors of changes in emotional intelligence

The regression model for EI gain among interns was statistically significant (*F* = 2.103, *p* = 0.050) and identified lower age and longer program (with those taking their CPE unit part-time over 20 or more weeks showing greater gains than those taking a shorter intensive or extended unit) as significant predictors of EI gain (*t* = −2.261, *p* = 0.026 and *t* = 2.806, *p* = 0.006, respectively). However, the model showed overall poor predictive capacity, accounting for only 6.6% of the variation in outcome (adjusted R^2^ = 0.066). This suggests that unknown variables not included in the regression may contribute more to EI gain than age and program length. The EI model for residents was not significant (*F* = 0.487, *p* = 0.815) and identified no individually significant predictors. Regression results for EI are summarized in Table 4, and each regression model is detailed in the Supplementary Tables 1–6.

**Table 4:** Summary of regression results for SSEIT and CASES Parts 1 and 2 gain.

Pre-post gain	R^2^	Significant predictors	*B*	SE	Std. *b*	*t*	*p*	95% CI Lower	95% CI Upper
Interns									
SSEIT	0.066	Age	−0.176	0.078	−0.222	−2.261	0.026	−0.331	−0.022
		Longer unit	7.033	2.506	0.282	2.806	0.006	2.062	12.004
CASES Part 1	0.166	Related experience^a^	−0.536	0.266	−0.209	−2.018	0.046	−1.062	−0.009
		Gain in EI	0.044	0.011	0.372	3.981	<0.001	0.022	0.067
CASES Part 2	0.242	Related experience^a^	−0.548	0.253	−0.213	−2.163	0.033	−1.051	−0.045
		Gain in EI	0.056	0.011	0.472	5.296	<0.001	0.035	0.078
Residents									
SSEIT	−0.044	No sig predictors							
CASES Part 1	0.229	Being male	−0.676	0.256	−0.288	−2.643	0.010	−1.187	−0.165
		Gain in EI	0.044	0.009	0.504	4.795	<0.001	0.026	0.063
CASES Part 2	0.049	Gain in EI^b^	0.009	0.003	0.355	2.802	0.007	0.002	0.015

a People with >3 years of prior experience related to spiritual care and counseling had smaller gains in outcome variable than people with <1 year of experience.

b Outcome log-transformed to meet normality requirements for linear regression.

SSEIT, Schutte Self-Report Emotional Intelligence Test (55, 56); CASES, Counselor Activity Self-Efficacy Scales (38).

### 3.5 Predictors of changes in counseling self-efficacy

For CASES Part 1: Helping Skills Self-Efficacy gain, regression models were statistically significant and reasonably predictive for both interns (*F* = 3.719, *p* < 0.001, adj. R^2^ = 0.166) and residents (*F* = 4.094, *p* < 0.001, adj. R^2^ = 0.229). In keeping with theory, greater gains in EI predicted greater gains in CASES Part 1 for both interns and residents (*t* = 3.981, *p* < 0.001 and *t* = 4.795, *p* < 0.001, respectively). In addition, CASES Part 1 gain was predicted by previous relevant experience among interns, with those having more than 3 years of relevant experience showing lower gains than those with less than 1 year of experience (*t* = −2.018, *p* = 0.046). It was also predicted by gender in residents, with those identifying as male having smaller gains than those identifying as female (*t* = −2.643, *p* = 0.010). *Post-hoc* analysis comparing baseline levels of the CASES Part 1 subscale shows that interns with greater than 3 years of relevant experience have a higher average item rating (*M* = 6.05 on a 1–9 scale, SD = 1.32) compared to those with less than 1 year of experience (*M* = 5.62, SD = 1.24). In addition, male residents and female residents have comparable baseline CASES Part 1 average item ratings (*M* = 6.13, SD = 1.51 for men, *M* = 6.06, SD = 1.40 for women). Table 4 presents the summary of regression results for CASES Part 1 and Part 2; detailed results of each regression are provided in the Supplementary Tables 1–6.

The regression for CASES Part 2: Session Management Self-Efficacy scale was significant for interns only, with reasonable predictive capacity (*F* = 5.348, *p* < 0.001, adj. R^2^ = 0.242). As with CASES Part 1, greater gains in EI predicted greater gains in CASES Part 2 (*t* = 5.296, *p* < 0.001). Furthermore, CASES Part 2 gains were lower for those with more than 3 years of relevant experience compared to those with less than 1 year of experience (*t* = −2.163, *p* = 0.033). Although the regression for CASES Part 2 was not significant overall among residents (*F* = 1.474, *p* = 0.195) and had poor predictive capacity (adj. R^2^ = 0.049), EI gain was identified as a significant predictor (*t* = 2.802, *p* = 0.007). Again, there appear to be unidentified variables that might contribute more predictive power to the outcome. Finally, we noted that the mode of CPE delivery—in-person, hybrid (mixed in-person and online), or online—did not predict increases in EI and CSE.

### 3.6 Correlations between emotional intelligence and counseling self-efficacy

Spearman’s correlation results indicated that the pre-post change in EI (overall SSEIT) was significantly and moderately associated with changes in CSE on the CASES Part 1: Helping Skills Self-Efficacy scale (Spearman’s Rho [r_s_] = 0.391) and the CASES Part 2: Session Management Self Efficacy scale (r_s_ = 0.445) (*p*-values < 0.001) in the entire sample (see Table 5).

**Table 5:** Spearman’s correlation between pre-to-post gain in emotional intelligence and counseling self-efficacy scales.

Pre-post gain	SSEIT	CASES Part 1	CASES Part 2
SSEIT	1	0.391*	0.445*
CASES Part 1		1	0.694*
CASES Part 2			1

* *p* < 0.001.

SSEIT, Schutte Self-Report Emotional Intelligence Test (55, 56); CASES, Counselor Activity Self-Efficacy Scales (38).

## 4 Discussion

We investigated the changes in emotional intelligence and counseling self-efficacy that occur when students participate (a) in their first stand-alone unit of CPE (i.e., interns) or (b) in a CPE residency program (i.e., residents). We found emotional intelligence and counseling self-efficacy significantly increased among both types of participants from the beginning to the end of their CPE, with effect sizes ranging from moderate to large. Although causal conclusions cannot be drawn, the results suggest that CPE fostered growth in EI and CSE over the course of training and that gaining EI contributed to enhanced CSE during CPE. Moderate-to-large effect sizes demonstrate practical significance, indicating meaningful, real-world improvements of EI and CSE during CPE beyond mere statistical significance. Our study’s multicenter design, combined with validated instruments, further strengthens the evidence for the impact of CPE as typically delivered across multiple CPE centers in the United States, thereby expanding findings from prior studies with a single center or small samples.

### 4.1 Gaining emotional intelligence

Our study’s results, showing significant improvements in EI in CPE programs, build on the findings of Jankowski et al. (20) and Szilagyi et al. (14), which demonstrated that participation in the CPE program led to increases in EI, as measured by SSEIT, at rates of growth of 2.2 and 7%, respectively. They are also consistent with existing qualitative findings from Vanderstelt et al. (21) and Szilagyi et al. (14), which described CPE’s impact in fostering students’ EI and their ability to attend to emotions in their clinical work and reflective practice. Generally, EI scores in our sample were in line with those found in other studies using the same instrument among other health professionals (34, 61, 62). Our findings support the idea that improving EI in CPE contributes to chaplains’ capacity for attending to complex and intense emotions in clinical situations (14, 26). Based on associations identified in other studies, the improvements in EI suggest that this growth over the course of a CPE program may also confer benefits relating to clinical decision-making (35), caring behavior (33), counseling self-efficacy (48, 49, 63), leadership (27), and work-related wellbeing and job satisfaction (30, 31, 33, 34, 36, 62, 64).

### 4.2 Gaining counseling self-efficacy

The finding that participants’ CSE significantly increased during CPE corroborates earlier findings on CPE’s role in developing students’ clinical, spiritual care, and counseling skills (14, 17, 18, 20). Rates of CSE increases in our study were comparable to those measured by the CASES instrument among trainees in CPE (14) and other health professions, such as counseling (38), social work (40), and psychology (47).

These results suggest that participants in CPE may acquire sufficient knowledge and practice that lead to emerging counseling skills and mastery experiences, a primary source of self-efficacy. Typically, these mastery experiences, combined with explicit feedback, observation and modeling, and proximal goals, then contribute to one’s self-efficacy, which is crucial for motivation, effort, and persistence in both learning and practice (46, 65). The curricular elements of CPE’s experiential and action-reflection learning model incorporate these key promoters of self-efficacy. It also highlights the fact that chaplains-in-training and practicing chaplains need motivation as well as skills to function effectively in their clinical roles; the development of CSE connects knowledge and skills to practice by fostering the motivation to apply what has been learned (65).

We observed different baseline scores and magnitudes of gain on the subscales of the CASES instrument. For example, at baseline and post-training follow-up, the participants’ highest CASES scores were on the Exploration Skills subscale. That is, while this measure was relatively high to begin with, our participants nonetheless experienced further growth in confidence in their Exploration Skills that include asking open-ended questions, listening, reflecting feelings, restating/rephrasing, and attending to their care recipients (38). On the other hand, our study’s participants reported the lowest baseline levels for Action Skills self-efficacy, which entails using more structured approaches such as providing direct guidance, information, or resources and trying on new behaviors or roles (38). While they made substantial strides, showing the highest rates of increase in this area, it remained the lowest subscale at post-training. A possible interpretation is that chaplaincy training and practice typically embrace non-directive supportive approaches, emphasizing exploration and listening (66).

### 4.3 Emotional intelligence contributed to counseling self-efficacy

Our analysis revealed that developing EI significantly enhanced CSE among interns and residents. The regression models showed that EI gains were significant predictors of CSE gains among interns and residents. In addition, we found significant correlations between EI and CSE changes in our sample. Notably, in our regression models, the increases in EI were the strongest predictor of increases in CSE. This finding supports the notion that increasing EI—the ability to perceive, understand, use, and manage emotions in self and others (25)—is interconnected with increasing CSE. A possible explanation might be that CPE learning helps participants translate their growing EI into helping and counseling skills. Finally, these findings are congruent with studies showing a positive relationship between EI and CSE (49, 63) and EI accounting for significant CSE variance in other health professions (48).

### 4.4 Longer learning experiences allowed greater growth

The EI and CSE gains of the residents surpassed those of the interns and did not reach a plateau at the midpoint of their residency; rather, they continued to rise through the completion of their programs. Although residents began at a similar baseline as interns, their rate of growth across three or four consecutive CPE units ultimately outpaced the gains achieved by participants in a single CPE unit, and despite residents beginning CPE at a higher CSE baseline, they still showed greater growth than interns. This result shows that those receiving a greater “dose” of CPE experienced greater growth in EI and CSE. Previous studies did not explore the effects of CPE residency programs over multiple CPE units or compare them to a stand-alone CPE unit. An exception is Fitchett and Gray (17), which included CPE residents and no interns, and reported significant growth in various competencies. An imperfect comparison point might be the Jankowski et al. (20) study that included students with 1, 2, or more previous CPE units but still measured changes over a single CPE unit. They found that having prior CPE units was associated with higher baseline pastoral skills and less gain in pastoral skills, while it was not associated with baseline or change in EI. In contrast, our findings indicate that residents—the majority of whom had at least one prior CPE unit—had higher baselines in CSE and attained larger gains in EI and CSE over multiple CPE units than interns completing one initial CPE unit.

The role of the length of the stand-alone CPE unit for interns is notable since regression results showed that those completing a CPE unit over more than 20 weeks gained more EI compared to those completing their one CPE unit in a more compressed timeframe. This suggests that the longer timespan allowed more time for the interns to improve and integrate their capacity to recognize, use, and manage emotions. In contrast with our results, O’Connor et al. (67) found that students in an intensive 11-week unit rated their personal growth, professional growth, and theological reflection at the end of their training slightly higher than students in a 6-month unit. However, this study did not test whether these differences were statistically significant in their small sample. Jankowski et al. (20) reported a significantly greater increase in EI among students in intensive, shorter units compared to those in extended, longer CPE units, the opposite of our findings. Further research is needed to clarify these divergent findings and better understand how the length of the single CPE unit influences EI.

### 4.5 Other predictors of changes

We examined possible predictors of change, including demographic factors, prior experience, and program delivery modality. We found that most of the variables tested did not predict differential gains in EI. Our analysis did not identify any significant predictors of EI gains among residents. Among interns, lower age predicted greater increases in EI, in addition to the length of the CPE unit discussed above. However, the model with these predictors explained little total variance in EI, suggesting that unknown variables not included in the regression may contribute more to EI gain.

Among interns, those with more than 3 years of relevant prior experience had smaller gains in CSE than those with less than 1 year experience, an understandable finding considering there were higher baseline scores (and therefore less room for improvement) among learners who entered their initial unit of CPE with more extensive spiritual care or counseling experience. Jankowski et al. (20) similarly reported that more experience in ministry was associated with higher pastoral skills at baseline and less change in those skills. Lent et al. (38) also found that participants with more than 3 years of counseling-related experience reported significantly higher CSE scores than those with less than 1 year.

Our study’s data collection period (July 2020–January 2022) coincided with the COVID-19 pandemic. The pandemic prompted a rapid pivot to remote learning in CPE (68) and led to examining the role of CPE delivery mode—in-person, hybrid/blended, and online—on the study outcomes. We found that the mode of delivery was not associated with the extent to which growth in EI and CSE was experienced in CPE. This finding is consistent with reports from educators and students who described online or hybrid modalities as equally effective as in-person CPE and not compromising the quality of learning while recognizing its challenges and limitations (14, 69, 70).

### 4.6 Practical implications

The practical implications and potential mechanisms behind our findings require further exploration. In addition to directly addressing emotions and helping and counseling skills, several pedagogical approaches within the CPE learning model work together to foster EI and, consequently, CSE. Group-based, interactive, and experiential learning activities create opportunities for EI development by helping students gain self-awareness, recognize and discuss their own and others’ emotions, receive and offer feedback on emotional and social dynamics in group and clinician interactions (e.g., verbatim clinical case discussions), and critically reflect on their professional growth and clinical practice. Supervised clinical practice, combined with ongoing feedback and reflection, enables students to apply their developing EI and counseling skills and gradually improve mastery as they manage emotions and relationships in clinical interactions.

These elements of CPE align with strategies that have been shown to increase EI in health professions education, as supported by research (71) and expert recommendations (72, 73). A recent systematic review emphasized the importance of incorporating experiential learning, reflective practices, narrative approaches, communication skills training, and patient interaction into educational efforts to develop EI (71). CPE students will benefit from further enhancing these areas of CPE to purposely foster EI and CSE. In addition, our results support the importance of providing sufficient educational time for students to achieve and integrate EI learning, as well as intentionally building on students’ prior experiences related to spiritual care and counseling.

### 4.7 Implications for future research

Future studies are needed to evaluate which instructional strategies are most effective in promoting EI and counseling skills in CPE and whether these gains are sustained over the long term. Additional research may help develop a clearer picture of the role that the length of the intern CPE unit plays in fostering EI, CSE, and other relevant outcomes. Future investigations can benefit from randomized or waitlist-controlled designs to increase the quality and generalizability of evidence. Using comparison groups would allow researchers to compare CPE’s effectiveness in producing the desired outcomes with that of other educational programs. Future research should also investigate center-level effects with a sufficiently large sample size, which may necessitate multiple years of data collection.

Further research is needed to better understand other key outcomes of CPE by measuring established constructs with validated instruments in large, multicenter student samples. This work should also involve either adapting existing instruments or developing new ones and validating them to measure chaplain competencies in educational and practice settings. Finally, there is abundant room for further progress in using research designs with observer-rated or simulation approaches and supervisor ratings, alongside self-rated tools, to assess the development of CPE students’ competencies.

### 4.8 Strengths and limitations

Our study’s strengths include a large sample with fairly diverse participants enrolled in multiple CPE programs geographically distributed across the United States. This strengthens the case that our results can be attributed to CPE itself as typically delivered rather than to one specific CPE program or educator. We measured our outcome variables, EI and CSE, with validated and widely used instruments, not yet commonly used in CPE research. We investigated the relationship between EI and CSE, connecting EI growth to practical helping and counseling skills. Together, these addressed several methodological shortcomings of previous CPE research.

The study design, using a pretest/posttest approach, has inherent limitations and is susceptible to selection bias and confounders. Our design did not include a control group and aims to examine center-level effects and differences. Although we had a strong (64%) initial survey response rate and retained 72% of the baseline sample, we are unable to presume that those who dropped out would have reported less or more gains in EI and CSE. Analysis comparing baseline characteristics of those who dropped out of the study and the pre-post sample was not conducted.

Self-reported measures can be associated with limitations due to multiple factors at play in self-assessments, such as cognitive processing, introspective ability, social desirability bias, and response bias. It is possible that participants’ investment in their learning, as well as their wish to be seen as having progressed, may have led to an overestimation when reporting their EI and CSE post-training. However, Urbani et al. (74) found the opposite when comparing self-rated and trained-raters’ assessments of counseling skills: counseling students overestimated their counseling skills compared to trained-raters at pre-training, but they estimated their counseling skills as lower than trained-raters at post-training. So, it is possible that participants in our study overestimated their EI and CSE levels at the start of the program and that growth in EI and CSE was actually higher than we measured. Finally, without long-term follow-up, we did not gather data on whether and for how long students’ gains in EI and CSE are sustained.

## 5 Conclusion

This study demonstrates that participants’ emotional intelligence and counseling self-efficacy significantly increased over the course of Clinical Pastoral Education. Both interns and residents exhibited important and practical gains in these areas, with residents showing continued growth throughout their programs. The research reveals a strong relationship between EI development and CSE improvement, suggesting that EI growth contributes to increased confidence in counseling abilities. Notably, longer CPE units facilitated greater EI gains among interns. The study’s results underscore CPE’s apparent effectiveness in fostering critical skills for spiritual care providers, irrespective of delivery modality or participant demographics. The findings highlight the importance of optimizing CPE curricula that center on experiential learning, interactive feedback, reflection, and supervised clinical practice. These elements are crucial for promoting self-awareness, social–emotional learning, and skill development. Efforts should emphasize creating gradual mastery experiences in low-stakes practice environments by using strategies such as role-play and simulation, which are essential for fostering self-efficacy. While this research advances our understanding of CPE outcomes, it also highlights the need for further investigation into specific instructional strategies, comparative effectiveness, and rigorous assessment methods to continue exploring and validating the impact of CPE in chaplaincy education.

## Data Availability

The datasets presented in this study can be found in online repositories. The names of the repository/repositories and accession number(s) can be found at: Harvard Dataverse https://doi.org/10.7910/DVN/WI6EWW.
