# Supplementary material for: Developing emotional intelligence and counseling self-efficacy in clinical pastoral education in healthcare settings: a multicenter pre-post study
Source: Front Med (Lausanne). 2025 Oct 10;12:1578653. doi: 10.3389/fmed.2025.1578653 (PMC12549629; doi:10.3389/fmed.2025.1578653)
Supplement: Supplementary file 1 [file Data_Sheet_1.pdf]

Supplementary Material

1 Supplementary Tables

Supplemental tables for the article titled "Developing emotional intelligence and counseling self-efficacy in clinical pastoral education in healthcare settings: A multicenter pre-post study."

Supplementary Table 1. Linear Regression on SSEIT Pre-Post Gain Across CPE Unit Among Interns

Predictors	B	SE	Std. β	t	p	95% CI Lower	95% CI Upper
(Constant)	12.019	4.329		2.777	0.007	3.433	20.605
Age	-0.176	0.078	-0.222	-2.261	**0.026**	-0.331	-0.022
Male	-0.588	2.022	-0.027	-0.291	0.772	-4.598	3.423
1-3 years related work experience[a]	-2.205	2.755	-0.087	-0.800	0.425	-7.669	3.260
>3 years related work experience[a]	-0.884	2.352	-0.041	-0.376	0.708	-5.549	3.782
Online program[b]	-0.531	2.442	-0.023	-0.218	0.828	-5.374	4.312
Hybrid program[b]	-0.780	2.671	-0.030	-0.292	0.771	-6.079	4.518
Part time (20 weeks or more)[c]	7.033	2.506	0.282	2.806	**0.006**	2.062	12.004

[a] Reference = <1 year prior experience relating to spiritual care and counseling
[b] Reference = In-person program
[c] Reference = Shorter duration CPE unit (<20 weeks)

Supplementary Table 2. Linear Regression on CASES Part 1 Pre-Post Gain Across CPE Unit Among Interns

Predictors	B	SE	Std. β	t	p	95% CI Lower	95% CI Upper
(Constant)	0.021	0.506		0.042	0.967	-0.983	1.026
Age	0.016	0.009	0.170	1.788	0.077	-0.002	0.034
Male	0.157	0.228	0.061	0.687	0.494	-0.296	0.609
1-3 years related work experience[a]	0.301	0.312	0.100	0.967	0.336	-0.317	0.920
>3 years related work experience[a]	-0.536	0.266	-0.209	-2.018	**0.046**	-1.062	-0.009
Online program[b]	-0.332	0.275	-0.121	-1.207	0.230	-0.879	0.214
Hybrid program[b]	0.253	0.301	0.082	0.838	0.404	-0.345	0.851
Part time (20 weeks or more)[c]	0.329	0.293	0.111	1.121	0.265	-0.253	0.911
Pre-Post Gain in SSEIT	0.044	0.011	0.372	3.981	**<.001**	0.022	0.067

[a] Reference = <1 year prior experience relating to spiritual care and counseling
[b] Reference = In-person program
[c] Reference = Shorter duration CPE unit (<20 weeks)

Supplementary Table 3. Linear Regression on CASES Part 2 Pre-Post Gain Across CPE Unit Among Interns

Predictors	B	SE	Std. β	t	p	95% CI Lower	95% CI Upper
(Constant)	0.435	0.483		0.900	0.370	-0.524	1.394
Age	0.008	0.009	0.088	0.968	0.335	-0.009	0.025
Male	0.293	0.218	0.113	1.344	0.182	-0.139	0.725
1-3 years related work experience[a]	0.139	0.298	0.046	0.467	0.642	-0.451	0.729
>3 years related work experience[a]	-0.548	0.253	-0.213	-2.163	**0.033**	-1.051	-0.045
Online program[b]	-0.244	0.263	-0.089	-0.929	0.355	-0.766	0.277
Hybrid program[b]	0.179	0.288	0.058	0.623	0.535	-0.391	0.750
Part time unit (20 weeks or more)[c]	0.276	0.280	0.093	0.986	0.327	-0.279	0.832
Pre-Post Gain in SSEIT	0.056	0.011	0.472	5.296	**<.001**	0.035	0.078

[a] Reference = <1 year prior experience relating to spiritual care and counseling
[b] Reference = In-person program
[c] Reference = Shorter duration CPE unit (<20 weeks)

Supplementary Table 4. Linear Regression on SSEIT Pre-Post Gain Across CPE Residency

Predictors	B	SE	Std. β	t	p	95% CI Lower	95% CI Upper
(Constant)	11.060	7.398		1.495	0.140	-3.706	25.825
Age	-0.163	0.142	-0.153	-1.144	0.257	-0.447	0.121
Male	2.998	3.363	0.112	0.891	0.376	-3.714	9.709
1-3 years related work experience[a]	4.214	5.314	0.144	0.793	0.431	-6.394	14.821
>3 years related work experience[a]	3.622	5.276	0.135	0.687	0.495	-6.908	14.152
Online program[b]	-1.699	5.090	-0.044	-0.334	0.740	-11.858	8.461
Hybrid program[b]	-0.873	3.462	-0.033	-0.252	0.802	-7.783	6.036

[a] Reference = <1 year prior experience relating to spiritual care and counseling
[b] Reference = In-person program

Supplementary Table 5. Linear Regression on CASES Part 1 Pre-Post Gain Across CPE Residency

Predictors	B	SE	Std. β	t	p	95% CI Lower	95% CI Upper
(Constant)	1.210	0.569		2.128	0.037	0.075	2.346
Age	0.012	0.011	0.124	1.069	0.289	-0.010	0.033
Male	-0.676	0.256	-0.288	-2.643	**0.010**	-1.187	-0.165
1-3 years related work experience[a]	-0.641	0.404	-0.250	-1.587	0.117	-1.447	0.166
>3 years related work experience[a]	-0.609	0.400	-0.258	-1.522	0.133	-1.409	0.190
Online program[b]	-0.235	0.385	-0.068	-0.609	0.544	-1.004	0.535
Hybrid program[b]	-0.041	0.262	-0.017	-0.156	0.877	-0.564	0.482
Pre-Post Gain in SSEIT	0.044	0.009	0.504	4.795	**<.001**	0.026	0.063

[a] Reference = <1 year prior experience relating to spiritual care and counseling
[b] Reference = In-person program

Supplementary Table 6. Linear Regression on CASES Part 2 Pre-Post Gain[a] Across CPE Residency

Predictors	B	SE	Std. β	t	p	95% CI Lower	95% CI Upper
(Constant)	2.649	0.173		15.324	0.000	2.303	2.995
Age	0.000	0.003	-0.004	-0.030	0.976	-0.007	0.007
Male	-0.128	0.082	-0.210	-1.561	0.124	-0.293	0.036
1-3 years related work experience[b]	-0.034	0.129	-0.051	-0.263	0.794	-0.291	0.224
>3 years related work experience[b]	-0.104	0.130	-0.169	-0.795	0.430	-0.364	0.157
Online program[c]	-0.034	0.129	-0.035	-0.264	0.793	-0.292	0.224
Hybrid program[c]	0.025	0.079	0.041	0.314	0.754	-0.134	0.183
Pre-Post Gain in SSEIT	0.009	0.003	0.355	2.802	**0.007**	0.002	0.015

[a] CASES Part 2 Pre-Post Gain was log-transformed to meet normality requirements for linear regression
[b] Reference = <1 year prior experience relating to spiritual care and counseling
[c] Reference = In-person program
